# EpCAM as a Novel Biomarker for Survivals in Prostate Cancer Patients

**DOI:** 10.3389/fcell.2022.843604

**Published:** 2022-04-20

**Authors:** Yang Liao, Mingxin Wu, Yingjie Jia, Ruiyu Mou, Xiaojiang Li

**Affiliations:** ^1^ Department of Oncology, First Teaching Hospital of Tianjin University of Traditional Chinese Medicine, Tianjin, China; ^2^ National Clinical Research Center for Chinese Medicine Acupuncture and Moxibustion, Tianjin, China

**Keywords:** EpCAM, ceRNA network, prognosis, immune infiltration, prostate cancer

## Abstract

**Background:** Due to the insufficient understanding of the biological mechanisms, the improvement of therapeutic effects of prostate cancer (PCa) is limited. There is an urgent need to find the molecular mechanisms and underlying PCa to improve its early diagnosis, treatment, and prognosis.

**Methods:** The mRNA expression profiles, survival and methylation data of PRAD were downloaded from The Cancer Genome Atlas (TCGA) database. The identification of differentially expressed genes (DEGs), Gene Ontology (GO) and Kyoto Encyclopedia of Genes and Genomes (KEGG) functional enrichment analyses were performed by R software. Subsequently, we identified the key gene and validated its prognostic role from the Human Protein Atlas (HPA) database, UALCAN and the LinkedOmics database. We performd correlation analysis and constructed the ceRNA network based on the data obtained from miRbase and starBase. Finally, we performed methylation analysis and evaluated the immune cell infiltration by Tumor Immune Estimation Resource (TIMER).

**Results:** A total of 567 DEGs were identified in PCa. ARHGEF38, SLPI, EpCAM, C1QTNF1, and HBB were regarded as target genes related to favorable overall survival (OS). Among them, EpCAM was considered as the most significant gene through the HPA database and receiver operating characteristic (ROC) analysis. A prognostic ceRNA network was constructed with EBLN3P, miR-204-5p, and EpCAM. EpCAM was found to be related to DNA methylation and tumor-infiltrating immune cells.

**Conclusion:** Our findings provide novel insights into the tumorigenesis mechanism of PCa and contribute to the development of EpCAM as a potential prognostic biomarker in PCa.

## 1 Introduction

Prostate cancer (PCa), one of the most common malignant tumors in male genitourinary system, tends to occur in elderly men. In 2020, there were about 1.4 million new cases of PCa worldwide, ranking second in male malignant tumors (Sung et al., 2021). Clinically, the majority of patients with PCa are diagnosed in the advanced stage due to the insidious development of tumor, which implies that early diagnosis of PCa to improve the prognosis is still at the initial stage of inquiry. Although the therapeutic landscape of non-metastatic PCa or castration-resistant prostate cancer (CRPC) has been transformed over the last decade by new therapeutics including chemotherapy and androgen deprivation therapy (ADT), therapeutic resistance, and tumor recurrence appear to be inevitable (Teo et al., 2019). Lethal metastatic PCa can exhibit heterogeneity at the genomic and phenotypic levels, posing obstacles for the diagnosis and treatment (Haffner et al., 2021). The insufficient understanding of the biological mechanisms inhibits the improvement of therapeutic effects. Hence, there is an urgent need to find the molecular mechanisms underlying PCa to improve its early diagnosis, treatment, and prognosis.

As a landmark cancer genomics program, the Cancer Genome Atlas (TCGA) database containing transcriptome profiling data and clinical data was often applied for identifying molecular biomarkers with prognostic significance in PCa. In a research, RNA sequencing (RNA-seq) and genome-wide methylome data from TCGA database were integrated to mine candidate driver genes involved in PCa development and progression, and Myeloid Ecotropic Insertion Site 2 (MEIS2) exhibited predictive significance in PCa patients (Nørgaard et al., 2019). Additionally, TCGA database exerted a prominent influence on redefining Inhibitor of Growth 3 (ING3) as an oncogene, which was opposed to the prevailing view that ING played an oncosuppressive role in PCa (Nabbi et al., 2017). In addition to typical functional genes, TCGA database was also used to screen the prognostic non-coding RNAs including DSCAM-AS1 and miR-17 (Dyson et al., 2018; Zhang et al., 2020).

As the most representative biomarker, the serum prostate-specific antigen (PSA) is extensively used clinically to screen for PCa. However, its specificity is limited because men with prostatic hypertrophy or prostatitis tends to perform high levels of PSA. A study indicated that PCA3 biomarker detection presented much more efficient than PSA detection currently used (Soares et al., 2019). Furthermore, there are still other clinically relevant biomarkers including prostate stem cell antigen (PSCA), prostate-specific membrane antigen (PSMA), prostatic acid phosphatase (PAP), prostate secretory protein-94 (PSP94), and circulating tumor cells. Unfortunately, their clinical application value and diagnostic accuracy need to be further evaluated to optimize patient’s therapy. Recently, a host of studies revolved around combined biomarkers which were considered to be potential strategies, with mixed results (Cucchiara et al., 2018). Hence, the identification of predictive biomarkers in the present case is necessary and challenging.

Long noncoding RNAs (lncRNA), as signals and guides in gene regulatory networks, can promote tumorigenesis and metastasis by alterations in their expressions and mutations (Wang and Chang, 2011; Bhan et al., 2017). In particular, lncRNAs mainly act as microRNA (miRNA) sponges to competitively bind to shared miRNA sequences, and participate in post-transcriptional, and translational regulation (Kopp and Mendell, 2018). Current studies indicated that a host of lncRNAs were involved in the occurrence and development of PCa by functioning as competing endogenous RNAs (ceRNAs) which were closely related to various biological processes (Wu et al., 2019; You et al., 2019). Thus, the molecular mechanisms of ceRNA provide a framework for the molecular studies to further dig the prognostic biomarkers.

**FIGURE 1:**
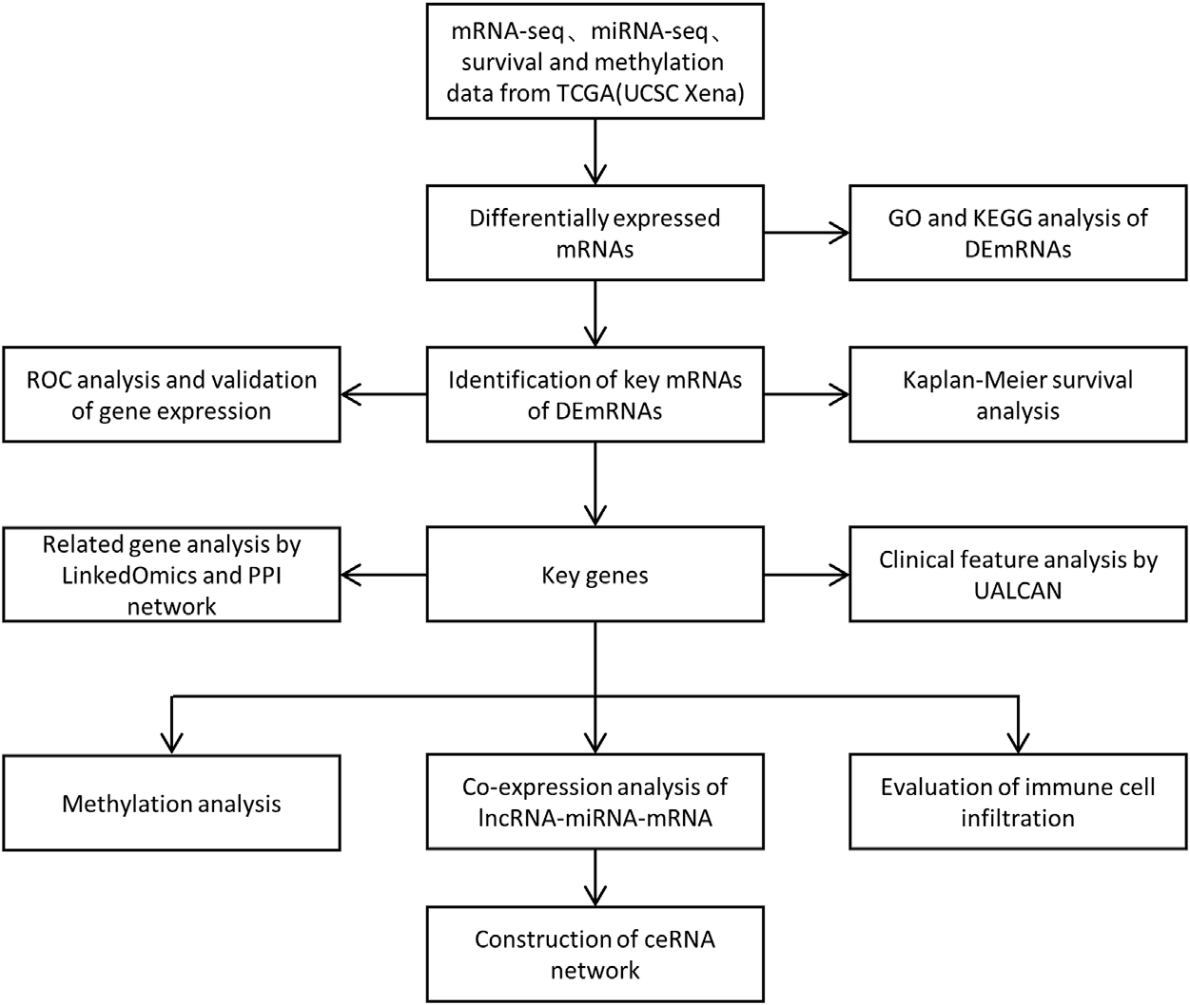
Flow chart for the identification and analysis of prognostic genes.

In the present study, we first performed differential expression analysis between normal tissues and PCa of TCGA database, and conducted the analysis of functional enrichment. In addition, we identified the target mRNA of differentially expressed mRNAs (DEmRNAs) by Kaplan-Meier survival analysis, ROC analysis and immunohistochemistry images obtained from the Human protein Atlas (HPA) database. Furthermore, we validated the prognostic role of the target gene in UALCAN, and constructed a PPI network based on String database. Eventually, we focused on the correlation between miRNAs, lncRNAs and the target mRNA, and established a ceRNA regulatory network based on the above results. We also performed the methylation analysis and evaluate the potential correlation between the target gene and immune infiltration levels in PCa (Figure 1). Our study aims to provide novel insights into the functional molecular in PCa, thereby revealing their potential prognostic value.

## 2 Methods

### 2.1 Data and Sources

The mRNA expression profiles, survival and methylation data of the PCa patients were downloaded from the TCGA data repository. UCSC Xena database (http://xena.ucsc.edu/) which contains normal prostate tissues and TCGA-PRAD tissues was utilized. We collected RNA-Seq data for 551 samples (499 tumor samples and 52 normal samples) in HTSeq-FPKM files, survival data for 623 samples in Survival Data files, mutation data for 484 samples in VarScan2 Variant Aggregation and Masking files, methylation data for 553 samples in Illumina Human Methylation 450 files. The miRNA expression profiles for 551 samples of the PCa patients were downloaded from TCGA database (https://cancergenome.nih.gov/). mRNA expression profiles of GSE46602, GSE32571 were downloaded from Gene Expression Omnibus (GEO). All the data above was annotated and consolidated by the performance of Perl script.

### 2.2 Differential Expression Analysis

Differentially expressed genes (DEGs) were identified by analyzing the normalized expression data of PCa and adjacent normal tissues using R package “limma”. |log2Fold change| ≥ 1 and False Discovery Rate (FDR) < 0.05 were considered statistically significant. Besides, univariate Cox regression analysis was performed for each gene to explore the correlation of gene expression with survival. All differentially expressed RNAs were visualized in volcano plot by R package “ggplot2”. For further analysis, Kaplan-Meier survival curves were performed to identify target genes on DEGs related to PCa prognosis. *p* < 0.05 was considered a threshold.

### 2.3 Functional Enrichment Analysis

Gene Ontology (GO) and Kyoto Encyclopedia of Genes and Genomes (KEGG) functional enrichment analyses were performed by R package “clusterProfiler” and “org.Hs.eg.db”. The cutoff criteria was adjusted *p*-value < 0.05. The circos plot was generated by R package “ggplot2”.

### 2.4 Prediction of the Key Gene and Clinical Correlation Analysis

The relationships between the expression of target genes and a series of clinical characteristics were analyzed by chi-square test. To screen the most significant genes related to prognosis in PCa patients, R package “timeROC” was used to perform receiver operating characteristic (ROC) curve. The HPA database (https://www.proteinatlas.org) was used to obtain immunohistochemistry images and corresponding data to validate gene expressions at the protein level (Uhlén et al., 2015). Once the key gene was identified, the online platform UALCAN (http://ualcan.path.uab.edu/) was applied to analyze the correlation between the key gene and clinical features (Chandrashekar et al., 2017). Besides, univariate and multivariate Cox regression analyses were performed to explore the correlation of the expression of key gene with OS among PCa patients. GEO and GEPIA databases were used to validate the differential expression of the key gene.

### 2.5 Identification of Related Gene

Related genes with the key gene were identified by the LinkedOmics database (http://www.linkedomics.org) (Vasaikar et al., 2018). To better understand the interactions of the key gene with related genes, we constructed a PPI network using the STRING online search tool (https://string-db.org/) (Szklarczyk et al., 2019). The median confidence score (0.4) was used to filter protein interactions. Cytoscape 3.9.0 was used to embody the constructed PPI network (Shannon et al., 2003).

### 2.6 Correlation Analysis of miRNAs-mRNAs-lncRNAs

All mature miRNA sequences were downloaded from the miRbase database (https://www.mirbase.org/) (Kozomara et al., 2019). The starBase (https://starbase.sysu.edu.cn/) (Li et al., 2014), DIANA-LncBase (http://www.microrna.gr/LncBase) (Karagkouni et al., 2020) and TargetScan (http://www.targetscan.org/) (Lewis et al., 2003) were used to predict the lncRNA–miRNA and the miRNA–mRNA interaction pairs, and the overlapping results were selected for further analysis. Pearson’s correlation was calculated to measure the expression correlation between miRNAs and mRNAs. We then conducted Pearson correlation analysis and selected the positive correlations between the lncRNAs and mRNAs. Pearson correlation coefficient < -0.2 and *p* < 0.01 were used as inclusion criteria.

### 2.7 Construction of ceRNA Network

Based on the above co-expression relationships, we selected lncRNA–miRNA–mRNA interaction pairs to build the ceRNA regulatory network which was visualized using Cytoscape 3.9.0. Furthermore, we identified miRNAs and lncRNAs significantly associated with patient prognosis, with the Kaplan-Meier curve analysis.

### 2.8 Evaluation of Immune Cell Infiltration and Methylation Analysis

Tumor Immune Estimation Resource (TIMER (https://cistrome.shinyapps.io/timer/); with Spearman’s method was used to determine the potential correlation between the key gene and tumor-infiltrating immune cells, including B cells, CD4^+^ T cells, CD8^+^ T cells, neutrophils, macrophages, and dendritic cells (Li et al., 2020a). In addition, associations between the key gene and gene markers of tumor-infiltrating immune cells were also explored. Finally, we assessed the correlation between methylation level of the key gene and its mRNA expression level.

## 3 Results

### 3.1 Differential Expression Analysis and Enrichment Analysis

**FIGURE 2:**
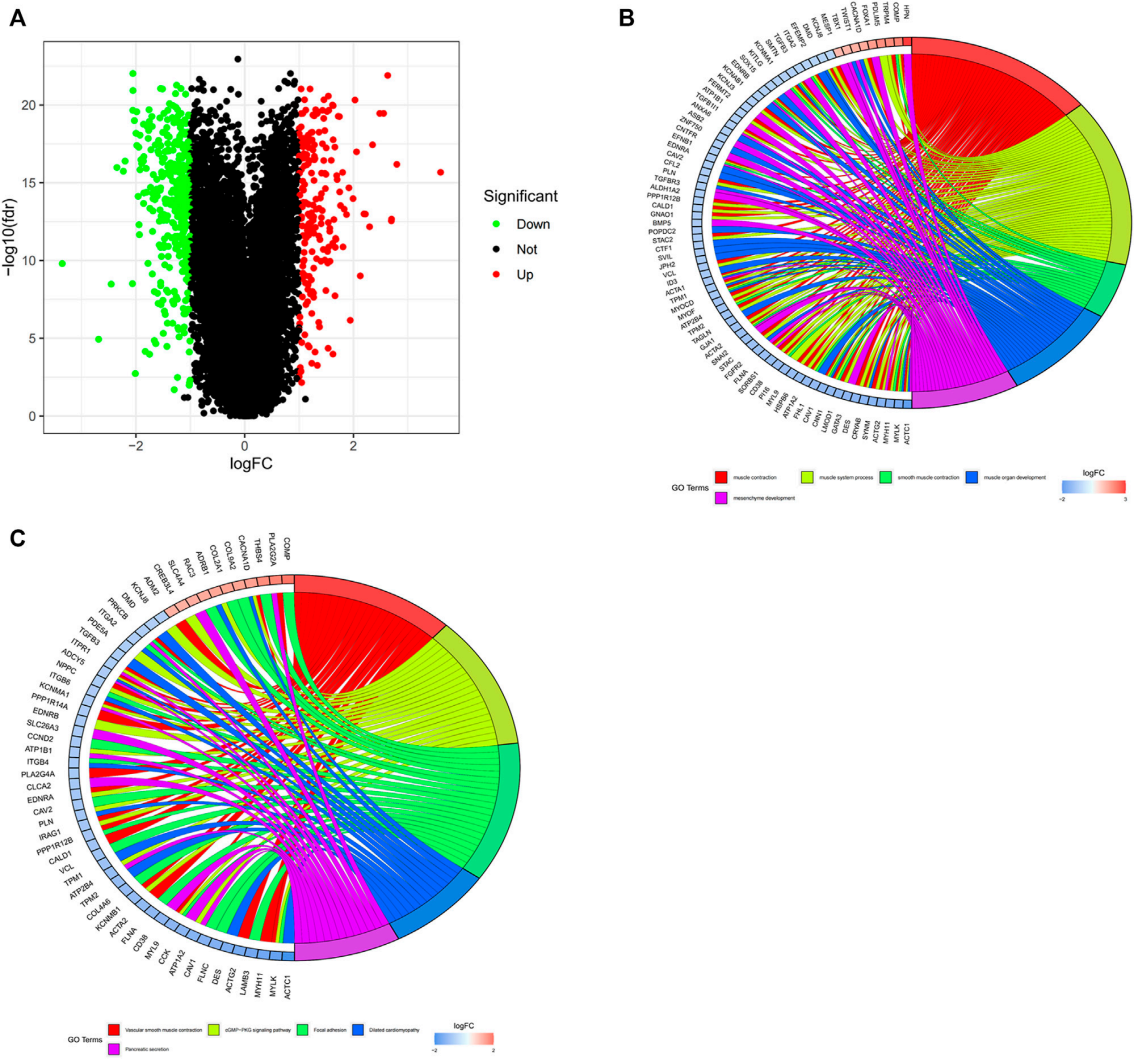
The identification and functional enrichment analysis of DEGs in PCa from TCGA database. **(A)** Identification of DEGs between normal and tumor tissues in PCa patients. Green and red indicate downregulated and upregulated genes, respectively. **(B,C)** GO and KEGG functional enrichment analysis of DEGs. Genes are shown on the left side of the chord diagram and enriched GO terms are shown on the right side. Red bars represent upregulated genes and blue bars represent downregulated genes.

The study aimed to explore the potential prognostic and therapeutic biomarkers of PCa. We obtained the expression profiles of mRNAs from TCGA database. The data was annotated and consolidated by the performance of Perl script. A total of 567 DEGs were selected from TCGA dataset, of which, 199 genes were upregulated and 368 genes were downregulated (Figure 2A). |log2Fold change| ≥ 1 and *p*-value < 0.05 were regarded as significant. GO and KEGG functional enrichment analysis were performed to distinct the functions of DEGs (Figures 2B,C). The results showed that the DEGs were associated with biological processes and signaling pathways, including muscle contraction, muscle system process, cGMP-PKG signaling pathway, and focal adhesion, etc.

### 3.2 Expression and Survival Analysis of the Target Genes

**FIGURE 3:**
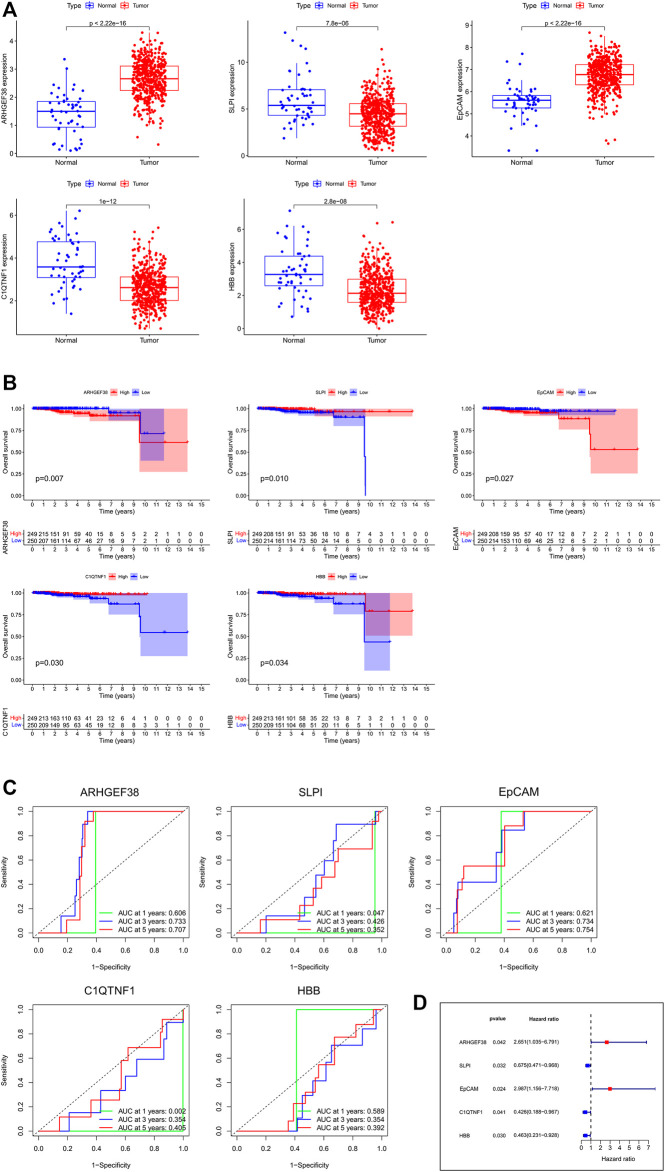
The identification and survival analysis of the most significant genes. **(A)** mRNA expressions between normal and tumor tissues in PCa. **(B)** Kaplan-Meier survival curves of target genes related to OS. **(C)** AUC of time-dependent ROC curves of target genes. **(D)** Forest plot of the risk scores about target genes.

In order to further identify the prognostic genes associated with PCa, Perl Script was used for exploring the most significant genes related to favorable overall survival (OS) of DEGs, and the screening criteria was *p* < 0.05. The screened genes included ARHGEF38, SLPI, EpCAM, C1QTNF1, and HBB (Supplementary Table S1). As shown in Figure 3A, ARHGEF38 and EpCAM were highly expressed in PCa tissues, while SLPI, C1QTNF1, and HBB were low expressed in PCa tissues. The association of the five genes with patient prognosis was analyzed using Kaplan-Meier survival curves. The results showed that SLPI, C1QTNF1, and HBB were positively correlated with OS, whereas ARHGEF38, EpCAM were negatively correlated with OS (Figure 3B). Meanwhile, the predictive performance of the risk score of key genes for OS was evaluated by time-dependent ROC curves. As the curve showed, among the five genes, the area under the curve (AUC) of EpCAM reached 0.621 at 1 year, 0.734 at 3 years, and 0.754 at 5 years (Figure 3C). Furthermore, the forest plot showed the hazard ratio of EpCAM (HR = 2.987, 95% CI: 1.156–7.718, and *p* = 0.024) was higher than other genes (Figure 3D).

**FIGURE 4:**
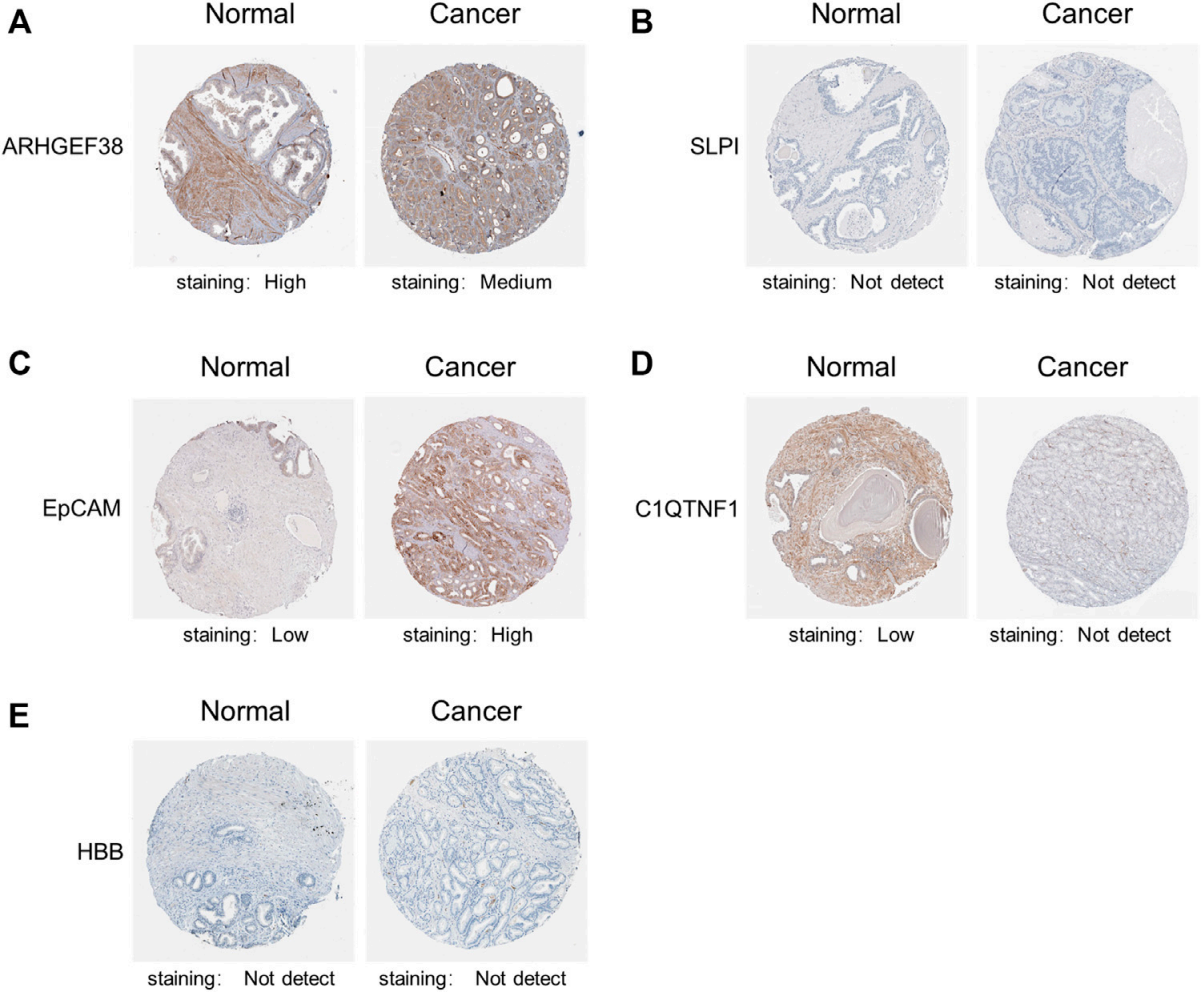
Representative immunohistochemistry images of the five genes in PCa tissues and normal prostate tissues (Human Protein Atlas). **(A)** High protein expression of ARHGEF38 was expressed in normal prostate tissues, while high protein expression of it was observed in PCa tissues. **(B,D,E)** SLPI, C1QTNF1, and HBB proteins were not expressed in PCa tissues, while low and no expressions of them were observed in normal prostate tissues. **(C)** Low protein expression of EpCAM was expressed in normal prostate tissues, while high protein expression of it was observed in PCa tissues.

Ultimately, we explore the protein expression patterns of these five genes in PCa by the HPA database. As shown in Figure 4, low protein expression of EpCAM was expressed in normal prostate tissues, while high protein expression of it was observed in PCa tissues (Figure 4C). Additionally, high protein expression of ARHGEF38 was expressed in normal prostate tissues, while high protein expression of it was observed in PCa tissues (Figure 4A). However, SLPI, C1QTNF1, and HBB proteins were not expressed in PCa tissues, while low and no expressions of them were observed in normal prostate tissues (Figures 4B,D,E). These results indicated that EpCAM expression was significantly related to prognosis of PCa patients, and can be exploited as a useful biomarker to predict PCa patients’ survival.

### 3.3 EpCAM Expression Is Significantly Associated With PCa

We implemented the chi-square test to study the detailed correlation of the expression of target genes with a panel of clinical features (Supplementary Table S2). As shown in Table 1, the expression of EpCAM was closely correlated with age (*p* = 0.029), Clinical T stage (*p* = 0.015), New tumor event after initial treatment (*p* = 0.037), and Prior malignancy diagnosis (*p* = 0.037). Besides, multivariate Cox regression analysis revealed that PSA level was significantly associated with survival (*p* < 0.05) (Supplementary Table S3).

**TABLE 1:** Correlation between EpCAM mRNA expression and clinicopathologic features in TCGA database.

Clinical features		EpCAM expression		*p* value
		Low (%)	High (%)	
Age	≤ 65	184 (66.9)	208 (75.4)	**0.029**
	> 65	91 (33.1)	68 (24.6)	
Race	White	230 (83.6)	241 (87.3)	0.176
	Black/African american	40 (14.5)	27 (9.8)	
	Asian	5 (1.8)	8 (2.9)	
Laterality	Bilateral	247 (89.8)	241 (87.3)	0.424
	Left	12 (4.4)	11 (4.0)	
	Right	16 (5.8)	24 (8.7)	
Biochemical recurrence	Yes	37 (13.5)	34 (12.3)	0.691
	No	238 (86.5)	242 (87.7)	
Clinical M stage	M0	272 (98.9)	275 (99.6)	
	M1	3 (1.1)	1 (0.4)	
Clinical T stage	T1	134 (48.7)	110 (39.9)	**0.015**
	T2	112 (40.7)	120 (43.5)	
	T3	27 (9.8)	45 (16.3)	
	T4	2 (0.7)	1 (0.4)	
Gleason score	6	27 (9.8)	24 (8.7)	0.298
	7	147 (53.5)	140 (50.7)	
	8	33 (12.0)	34 (12.3)	
	9	67 (24.4)	75 (27.2)	
	10	1 (0.4)	3 (1.1)	
New tumor event after initial treatment	YES	40 (14.5)	59 (21.4)	**0.037**
	NO	235 (85.5)	217 (78.6)	
Pathologic N stage	N0	233 (84.7)	218 (79.0)	0.080
	N1	42 (15.3)	58 (21.0)	
Pathologic T stage	T2	121 (44.0)	101 (36.6)	0.073
	T3	148 (53.8)	167 (60.5)	
	T4	6 (2.2)	8 (2.9)	
Radiation therapy	YES	32 (11.6)	45 (16.3)	0.114
	NO	243 (88.4)	231 (83.7)	
Prior malignancy diagnosis	YES	9 (3.3)	20 (7.2)	**0.037**
	NO	266 (96.7)	256 (92.4)	
PSA	≤10	268 (97.5)	262 (94.9)	0.121
	>10	7 (2.5)	14 (5.1)	

The bold values are statistically significant (*p* < 0.05).

**FIGURE 5:**
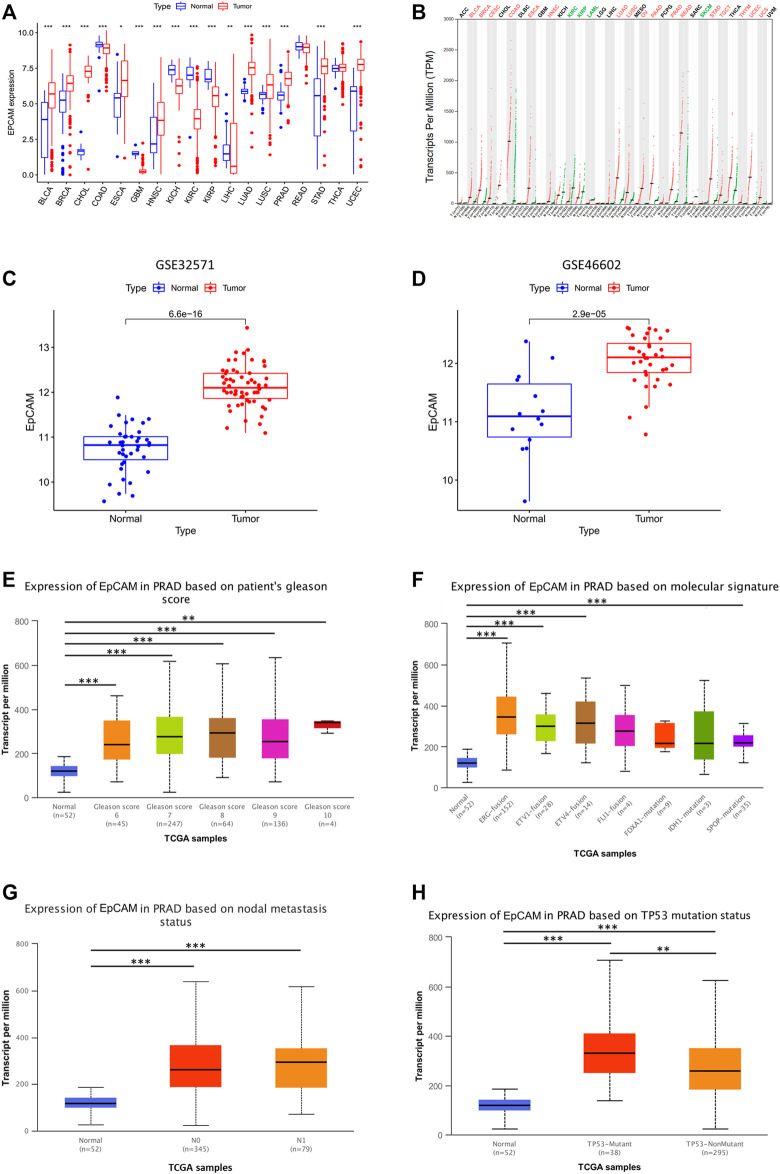
Validation of EpCAM expression and its correlation with clinical-pathological parameters in PCa patients. **(A)** EpCAM expression in multiple tumor tissues. **(B)** EpCAM expression in GEPIA database. **(C,D)** EpCAM expression in GSE46602, GSE32571 from GEO database. **(E–H)** Relationship between EpCAM expression and patients’ gleason score, molecular signature, nodal metastasis or TP53 mutation status. *p < 0.05, **p < 0.01, and ***p < 0.001.

We further evaluated the mRNA expression of EpCAM in multiple tumor tissues. As shown in Figure 5A, the mRNA expression of EpCAM was upregulated in BLCA, BRCA, CHOL, PRAD, etc, and downregulated in COAD, GBM, KICH, and KIRC, etc.

We used GSE46602, GSE32571, and GEPIA database for further validation, and the results showed that EpCAM expression was differentially expressed in these databases (Figures 5B–D). Moreover, we analyzed the relationship between EpCAM mRNA expression with clinical-pathological parameters of PCa patients by UALCAN, including patients’ gleason score, molecular signature, nodal metastasis, and TP53 mutation status. The results showed that the mRNA expression of EpCAM was remarkably correlated with patients’ gleason score, and patients who were with higher gleason score tended to express more significant mRNA expression of EpCAM (Figure 5E). In addition, the mRNA expression of EpCAM was closely related to molecular signatures, such as ERG-fusion, ETV1-fusion, ETV4-fusion, and SPOP-mutation (Figure 5F). However, nodal metastasis status was unrelated to the mRNA expression of EpCAM (Figure 5G). We then found that TP53 mutation was significantly correlated with the mRNA expression of EpCAM. As shown in Figure 5H, PCa samples with TP53 mutation had higher mRNA expression of EpCAM compared with those without TP53 mutation.

### 3.4 Gene Expression Correlation Between EpCAM and Related Genes

**FIGURE 6:**
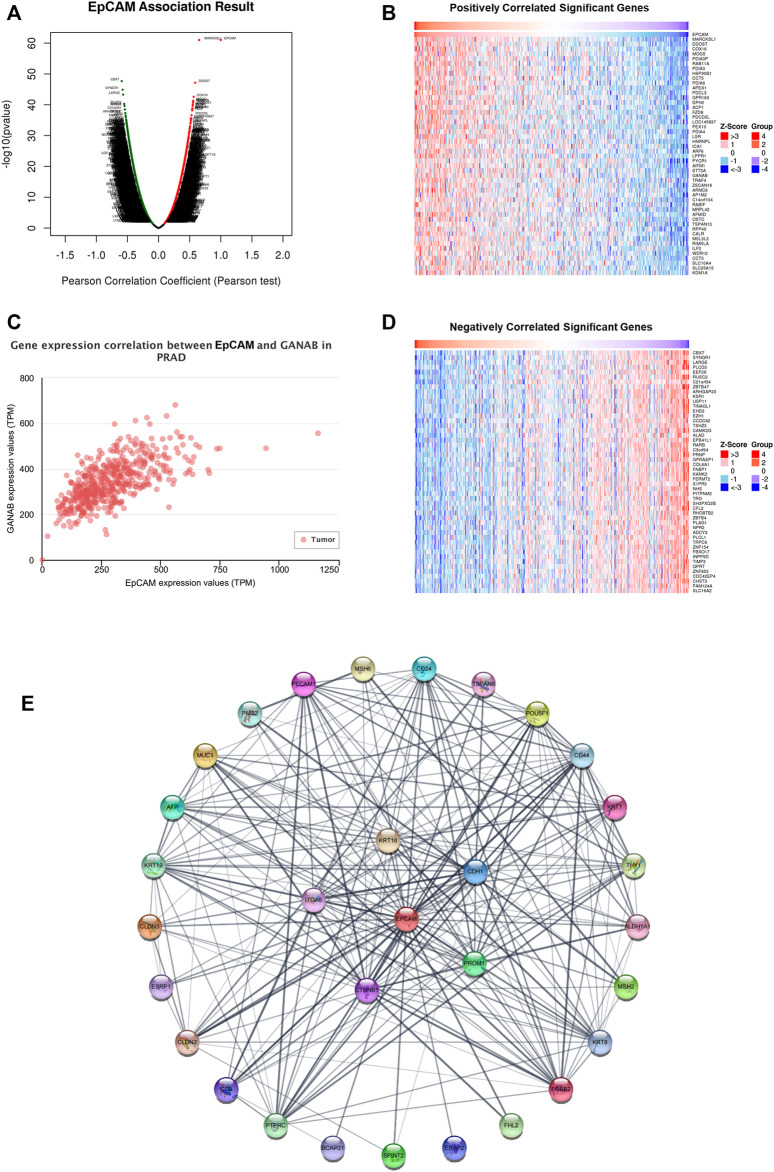
Gene expression correlation between EpCAM and related genes in PCa. **(A)** Volcano plot of significant genes correlated with EpCAM expression. **(B,D)** Heatmap of significant genes positively or negatively correlated with EpCAM expression. **(C)** GANAB was most positively correlated with EpCAM expression. **(E)** PPI network of 30 neighbor genes which were significantly associated with EpCAM. Each node corresponds to a protein-coding gene.

The volcano plot and heatmaps showed significant genes that were positively and negatively correlated with the mRNA expression of EpCAM (Figures 6A,B,D). As shown in Figure 6C, in PCa tissues, GANAB is most positively correlated with the mRNA expression of EpCAM. Moreover, we further analyzed 30 neighbor genes which were significantly associated with EpCAM and constructed a PPI network based on the STRING database (Supplementary Table S4). As shown in Figure 6D, the cadherin binding related genes including CTNNB1, CDH1, ITGA6, KRT18, and PROM1 were significantly associated with EpCAM.

### 3.5 Correlation Analysis of EpCAM and miRNAs

**FIGURE 7:**
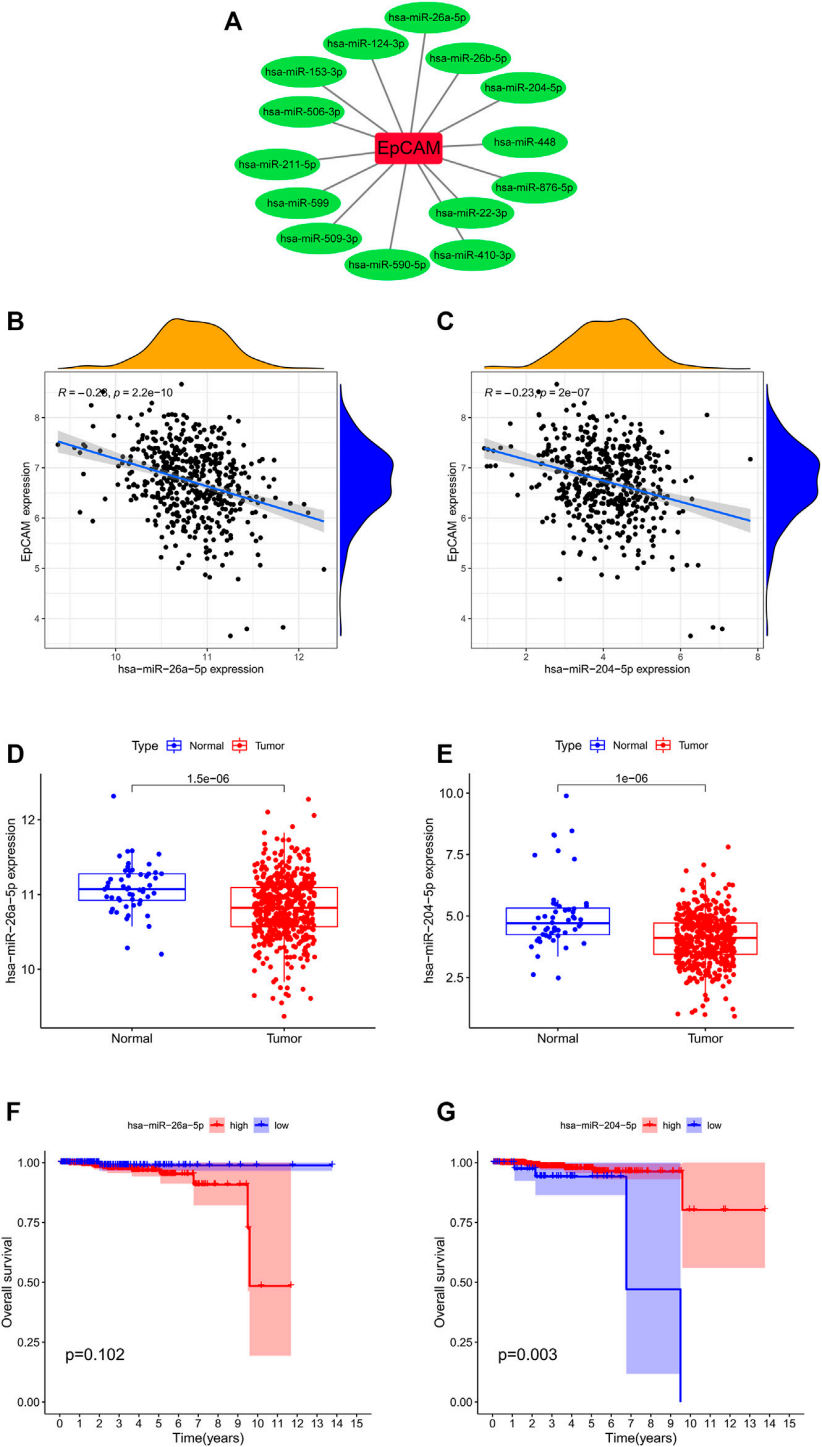
Correlation analysis of EpCAM and target miRNAs. **(A)** EpCAM was closely associated with 14 miRNAs. **(B,C)** The co-expression analysis of EpCAM and target miRNAs. **(D,E)** The expressions of target miRNAs in PCa tissues and normal prostate tissues. **(F,G)** Kaplan–Meier curve analysis of target miRNAs for the overall survival in PCa patients.

The miRNA-mRNA interactions were obtained from starBase, and TargetScan databases, and Figure 7A showed that EpCAM was closely associated with 14 miRNAs. After analyzing the co-expression, survival status and differential expression of these miRNAs, the results indicated that miR-26a-5p and miR-204-5p were negatively correlated with EpCAM expression. Furthermore, down-expression of miR-26a-5p was found in PCa tissues compared with normal tissues, while the expression of miR-204-5p increased in PCa samples. As shown in Figures 7F,G, higher expression of miR-204-5p was significantly associated with longer OS of PCa patients, while the expression of miR-26a-5p was unrelated to OS.

### 3.6 Construction of Prognosis-Related ceRNA Network

**FIGURE 8:**
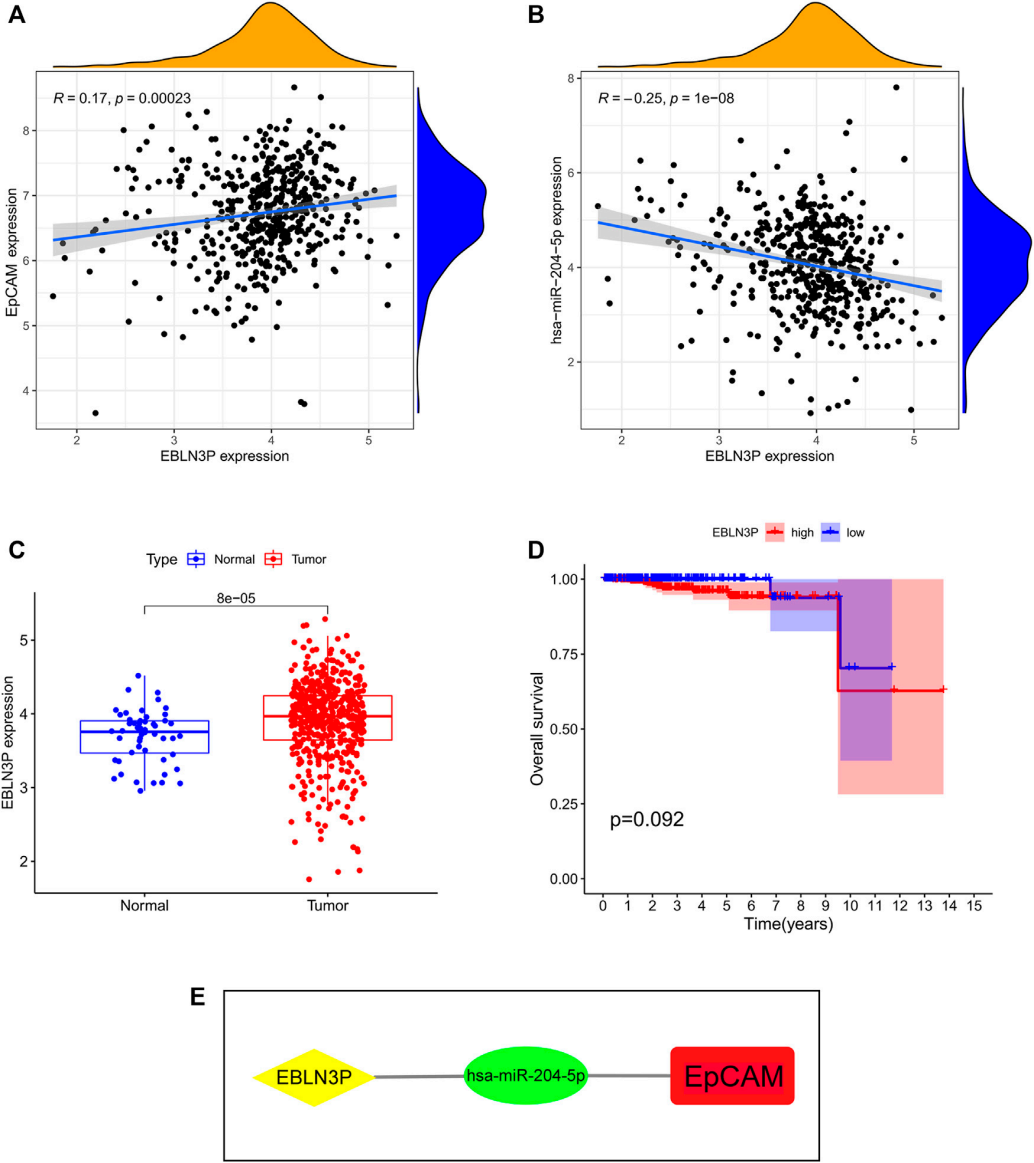
Identification of key lncRNA and construction of prognosis-related ceRNA network. **(A)** EBLN3P was positively correlated with miR-204-5p. **(B)** EBLN3P was negatively correlated with EpCAM. **(C)** The expression of EBLN3P in PCa tissues and normal prostate tissues. **(D)** Kaplan–Meier curve analysis of EBLN3P for the overall survival in PCa patients. **(E)** The ceRNA network of EBLN3P.

To verify the hypothesis that lncRNA positively regulates mRNA expression by interacting with miRNA, we obtained the miRNA-lncRNA interactions from starBase and DIANA-LncBase databases and performed Pearson correlation analysis between relevant lncRNAs and miR-204-5p. The results showed that EBLN3P was positively correlated with miR-204-5p (Figure 8A). Additionally, EBLN3P was negatively correlated with EpCAM (Figure 8B). As shown in Figure 8C, the expression level of EBLN3P was upregulated in PCa tissues compared with normal tissues. However, the expression level of EBLN3P showed no correlation with prognosis of PCa patients ((Figure 8D). As a result, we established the ceRNA network of EBLN3P (Figure 8E). Based on the ceRNA hypothesis, EBLN3P may upregulate the expression groups of EpCAM by acting as a sponge of miR-204-5p.

### 3.7 Methylation Analysis in PCa

**FIGURE 9:**
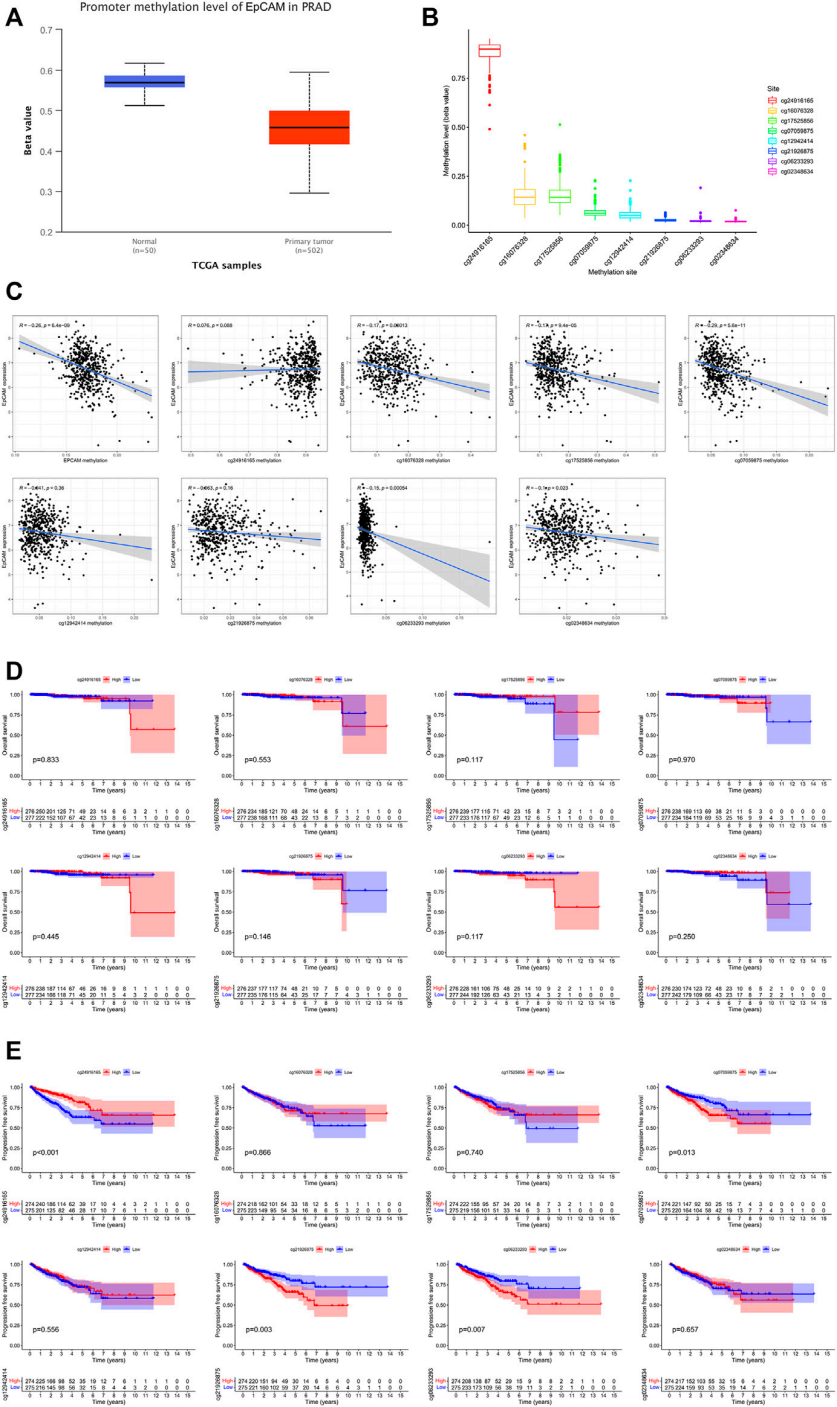
EpCAM DNA methylation analysis in PCa. **(A)** The methylation of EpCAM in PCa tissues and normal tissues. **(B)** Methylation level of EpCAM DNA CpG sites in PCa. **(C)** The correlation of EpCAM expression with methylation of EpCAM DNA CpG sites. **(D,E)** Kaplan-Meier curves of low and high methylation of EpCAM DNA CpG sites in PCa patients.

As exhibited in Figure 9A, we could observe that the level of EpCAM DNA methylation was higher in normal prostate tissues than that in PCa tissues. The distribution of eight EpCAM CpG sites was clearly exhibited in Figure 9B. Then we identify the EpCAM CpG sites at which methylation was most strongly correlated with the expression of EpCAM using Pearson correlation analysis. As shown in Figure 9C, we could observe a strong negative correlation between EpCAM expression and EpCAM DNA methylation. Five CpG sites (cg16076328, cg17525856, cg07059875, cg06233293, and cg02348634) were well negatively correlated with the expression of EpCAM. Ultimately, we performed Kaplan-Meier curve analysis of these selected EpCAM DNA CpG sites. As shown in Figure 9E, higher expression of 3 CpG sites (cg07059875, cg21926875, and cg06233293) were significantly associated with longer PFS of PCa patients, while lower expression of cg24916285 was related to longer PFS. However, the expression of EpCAM DNA CpG sites showed no correlation with OS of PCa patients.

### 3.8 Relationships of EpCAM With Tumor-Infiltrating Immune Cells

**FIGURE 10:**
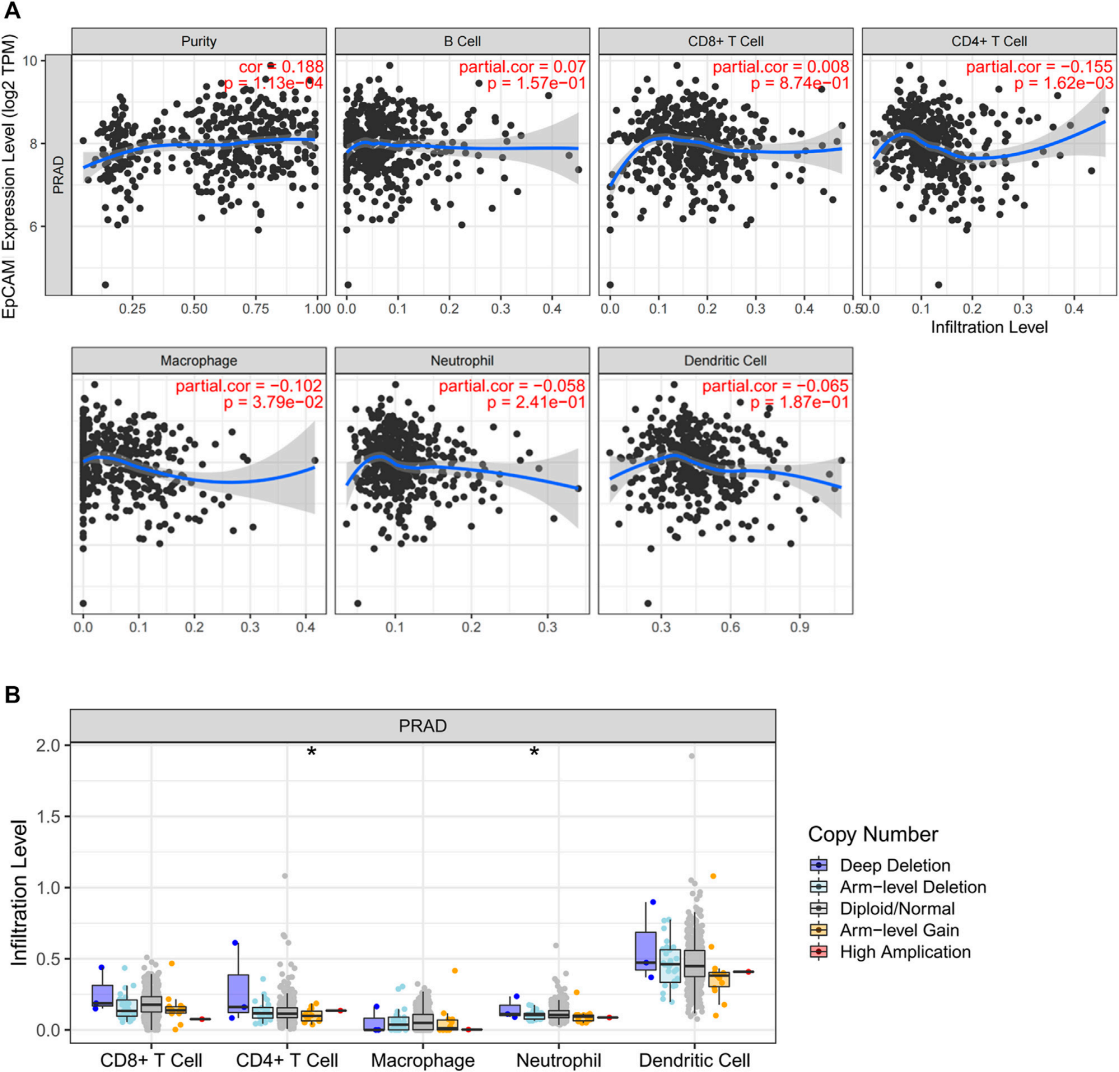
Correlation analysis between EpCAM expression and immune cells in PCa. **(A)** Scatterplots of correlations between EpCAM expression and immune cells, including CD4^+^ T cell, macrophage, B cell, CD8^+^ T cell, neutrophil or dendritic cell. **(B)** Correlation between somatic copy number variation and immune infiltration levels of five immune cells in PCa.

The TIMER database was applied to estimate the correlations of EpCAM expression with immune cell infiltration. As illustrated in Figure 10A, the expression of EpCAM was weakly correlated with immune infiltration of CD4^+^ T cell and macrophage. No association was revealed between EpCAM expression and B cell, CD8^+^ T cell, neutrophil or dendritic cell. We then explored the comparison of tumor infiltration levels in PCa with different somatic copy number alterations for EpCAM. The results showed that arm-level gain in PRAD was significantly associated with the infiltration of CD4^+^ T cells (*p* < 0.05). Moreover, arm-level deletion was related to the level of dendritic cell infiltration (*p* < 0.05) (Figure 10B).

**FIGURE 11:**
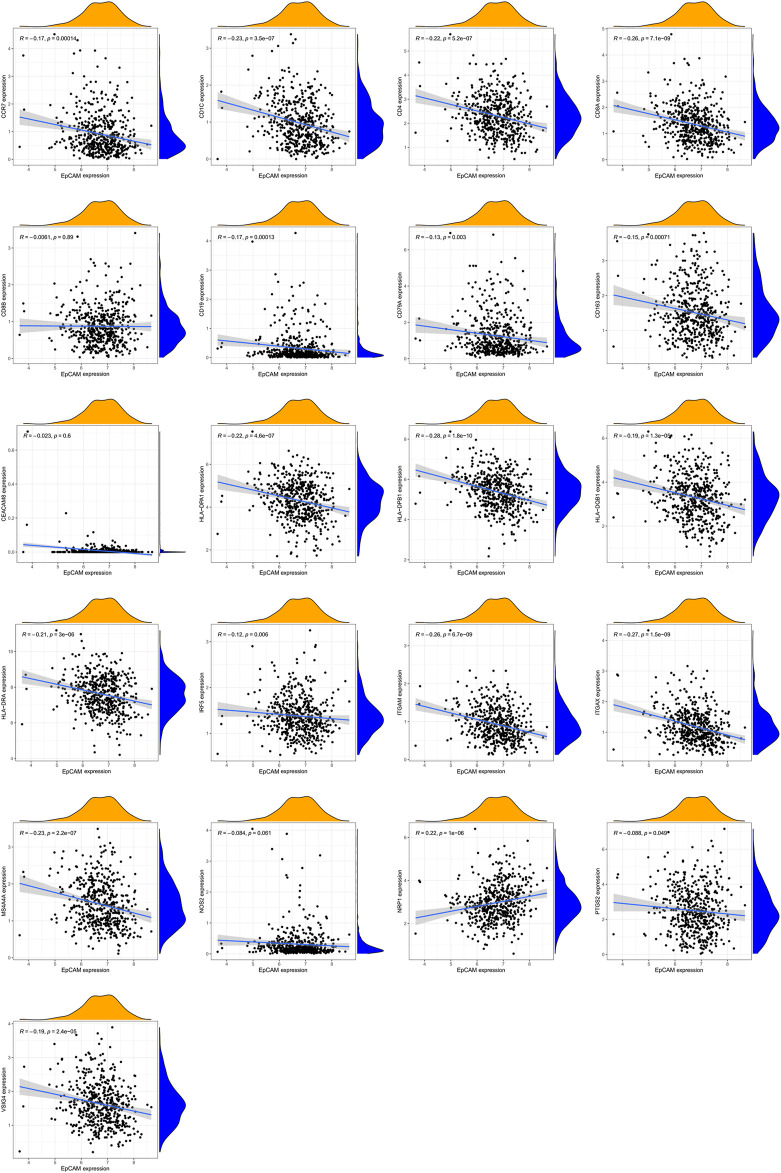
The mRNA expression of EpCAM was related to gene markers of immune cells.

Additionally, we further analyzed the correlations of EpCAM expression with the markers of 6 immune cells in PCa (Supplementary Table S5). These results based on tumor purity, revealing a significant correlation between EpCAM expression, and B cell markers (CD19, CD79A), CD8^+^ T cells markers (CD8A, CD8B), CD4^+^ T cell markers (CD4), M1 macrophage markers (NOS2, IRF5, and PTGS2), M2 macrophage markers (VSIG4, MS4A4A), neutrophil markers (CEACAM8, ITGAM, and CCR7) and dendritic cell markers (HLA-DPB1, HLA-DQB1, HLA-DRA, HLA-DPA1, CD1C, NRP1, and ITGAX) in PCa. As shown in Figure 11, no correlation was observed between EpCAM expression and CD8B, CEACAMB, and NOS2. In the remaining markers, EpCAM expression was negatively correlated with them except NRP1.

## 4 Discussion

EpCAM (epithelial cell adhesion molecule), as a Type I transmembrane superficial glycoprotein antigen, is expressed on the surface of basolateral membrane of multiple epithelial cells (Huang et al., 2018). In 1979, EpCAM was discovered as a tumor antigen expressed on colorectal carcinoma cells (Herlyn et al., 1979). As a multi-functional transmembrane protein, EpCAM is involved in the regulation of cell adhesion, stemness, proliferation, and epithelial-to-mesenchymal transition (EMT) of carcinoma cells. In order to achieve these functions, EpCAM plays an important role in signal transduction, and follows regulated intramembrane proteolysis to produce functionally active extracellular and intracellular fragments (Gires et al., 2020). Despite a flurry of related studies that have shown positive or negative links between EpCAM and tumor progression, few studies involve the specific cellular mechanism of EpCAM and its functional consequences (Fagotto and Aslemarz, 2020). Recent studies demonstrated that EpCAM was associated with therapeutic resistance, lymph node and bone metastases in PCa, but the direct molecular mechanism remains elusive (Campos et al., 2016; Ni et al., 2018). In this study, the results revealed that EpCAM is highly expressed in several types of cancer, with a particularly strong correlation between high EpCAM expression and a poor prognosis in PCa patients. Combined with the validation of immunohistochemical images from HPA database and Kaplan-Meier analysis, EpCAM could be a valuable prognostic biomarker worthy of further research in PCa.

In this report, we analyzed the expression of EpCAM in 33 different types of cancer through TCGA database, revealing the obvious difference of EpCAM expression between tumor and normal tissue in many cancers. EpCAM expression was upregulated in BLCA, BRCA, CHOL, and PRAD, etc., and downregulated in COAD, GBM, KICH, and KIRC, etc., which indicated that EpCAM was closely related to the occurrence and development of tumors. Additionally, based on the LinkedOmics and STRING database, we explored the correlation between EpCAM, and other genes and constructed a PPI network containing 30 neighbor genes. Obviously, the cadherin binding related genes including CTNNB1, CDH1, ITGA6, KRT18, and PROM1 were significantly associated with EpCAM, which also implied that EpCAM could play a role in tumor metastasis. Furthermore, elevated EpCAM levels closely related to TP53 mutation. Previous studies pointed out PCa with loss of the potent tumor suppressor TP53 exhibit poor outcomes and promote resistance to a spectrum of therapeutics (Hamid et al., 2019; Nyquist et al., 2020). Hence, EpCAM may be a potential target for interaction mechanism research to explain the inactivation or loss of TP53 in aggressive PCa.

Previous studies have confirmed that several cirRNAs or lncRNAs as the ceRNA facilitates prostate cancer progression (Huang et al., 2019; Yan et al., 2020). We analyzed the clinical and prognostic role of EpCAM in PCa patients and constructed a ceRNA network to explore the potential function of EpCAM and its related non-coding RNAs. In particular, based on the miRNA-lncRNA-mRNA co-expression relationship, we discovered that EBLN3P and miR-204-5p strongly correlated with EpCAM were potential to be prognostic biomarkers and therapeutic targets in PCa. Some studies have demonstrated that EBLN3P promotes the progression of liver cancer via alteration of microRNA-144-3p/DOCK4 pathway, and osteosarcoma through modifying the miR-224-5p/Rab10 signaling axis (Li et al., 2020b; Dai et al., 2021). Unfortunately, no research explores the correlation between prostate cancer and EBLN3P. Recent studies have indicated that miR-204-5p can promote apoptosis and block bone metastasis via inactivating NF-κB signaling in PCa (Lin et al., 2017; Wa et al., 2019), which is relevant to our analysis. The role of miR-204-5p in PCa involves lncRNA NEAT1 and upregulated ACSL4 expression, thus promoting the docetaxel resistance, cell proliferation, and invasion (Jiang et al., 2020). Importantly, Kaplan-Meier survival curve implied that high expression of miR-204-5p was truly associated with favorable OS among PCa patients. It is well established that EpCAM is an anchor molecule on circulating tumor cells (CTCs) which present the major source for metastatic cancer cells, and thus EpCAM may predict metastasis in some extent (Huang et al., 2018). Remarkably, UALCAN analysis demonstrated that elevated EpCAM level was correlated with high gleason score and nodal metastasis. These results suggested that EpCAM could be an indicator for metastasis and high grade malignancy of PCa. Therefore, it is possible that the novel ceRNA regulatory subnetwork could provide related molecules evaluated in combination with EpCAM, which could achieve a higher prediction performance of PCa metastasis.

It is becoming increasingly clear that immune cells as important mediators can be recruited into the prostate microenvironment. Hence, this function of immune cells in PCa is conducive to emergence of a multitude of clinical treatments, including checkpoint inhibitors and monoclonal antibodies (Strasner and Karin, 2015). Subsequently, we mined the TIMER database to explore the correlation between the expression of EpCAM and tumor-infiltrating immune cells including B cell, CD4^+^ T cell, CD8^+^ T cell, macrophage, neutrophil, and dendritic cell. Our results revealed that EpCAM expression was moderately negatively correlated with the degree of CD4^+^ T cell infiltration, and no correlations were observed between remaining immune cells and EpCAM. We further found that EpCAM expression was correlated with certain immunological markers including CD163, CCR7, CD1C, and NRP1, etc., which strongly suggested that EpCAM can regulate immune cell infiltration and activation in PCa. Infiltrating CD163‐positive M2 macrophages at a high level in the prostate tumor environment increases risk of dying of PCa (Erlandsson et al., 2019). Furthermore, TNF-α leads to the induction of CCR7 expression and the CCL21/CCR7 axis may increase the metastatic potential of prostate cancer cells in lymph node metastasis (Maolake et al., 2018). All in all, results above underlined that EpCAM could regulate immune cell recruitment in the immunological interactions in PCa, making it a valuable biomarker worthy of further research.

It is worth noting that several inevitable limitations exist in our study. First, the data we used were mainly obtained from TCGA database, therefore we could not validate the prognostic role of EpCAM from other databases. Second, the number of normal samples was small compared with tumor samples, and we need larger number of paired samples for further analysis. Finally, the detailed mechanism related to tumor development and the potential biological function of EpCAM need to be further verified by systematic experimental studies.

## 5 Conclusion

EpCAM is significantly upregulated in PCa and closely associated with PCa prognosis. Although further systematic experimental studies are required, our findings provide novel insights into the tumorigenesis mechanism of PCa and contribute to the development of EpCAM as a potential prognostic biomarker in PCa.

## Data Availability

The datasets presented in this study can be found in online repositories. The names of the repository/repositories and accession number(s) can be found in the article/Supplementary Material.
