# Supplementary material for: EpCAM as a Novel Biomarker for Survivals in Prostate Cancer Patients
Source: Front Cell Dev Biol. 2022 Apr 20;10:843604. doi: 10.3389/fcell.2022.843604 (PMC9065552; doi:10.3389/fcell.2022.843604)
Supplement: Supplementary file 1 [file Table4.DOC]

**Table S4. The correlation score between EpCAM and neighboring genes**

| Node1 | Node2 | Correlation score |  | Node2 | Correlation score |
| --- | --- | --- | --- | --- | --- |
| EpCAM | CLDN7 | 0.996 |  | CLDN3 | 0.772 |
|  | CTNNB1 | 0.959 |  | ITGA6 | 0.766 |
|  | FHL2 | 0.929 |  | ERBB2 | 0.753 |
|  | CDH1 | 0.916 |  | PMS2 | 0.740 |
|  | KRT19 | 0.913 |  | ESRP1 | 0.738 |
|  | CD44 | 0.897 |  | AFP | 0.722 |
|  | PROM1 | 0.857 |  | MUC1 | 0.721 |
|  | CD9 | 0.848 |  | ALDH1A1 | 0.719 |
|  | CD24 | 0.845 |  | MSH6 | 0.716 |
|  | PTPRC | 0.835 |  | POU5F1 | 0.716 |
|  | TSPAN8 | 0.825 |  | KRT7 | 0.711 |
|  | SPINT2 | 0.821 |  | MSH2 | 0.710 |
|  | KRT8 | 0.816 |  | THY1 | 0.707 |
|  | KRT18 | 0.787 |  | ERAP2 | 0.705 |
|  | BCAP31 | 0.779 |  | PECAM1 | 0.703 |
