# Supplementary material for: EpCAM as a Novel Biomarker for Survivals in Prostate Cancer Patients
Source: Front Cell Dev Biol. 2022 Apr 20;10:843604. doi: 10.3389/fcell.2022.843604 (PMC9065552; doi:10.3389/fcell.2022.843604)
Supplement: Supplementary file 2 [file Table3.DOC]

**Table S3. Univariate and multivariate analysis of the correlation of EpCAM expression with OS among PCa patients**

| Parameter | Univariate analysis | | | Multivariate analysis | | |
| --- | --- | --- | --- | --- | --- | --- |
|  | HR | 95% CI | P value | HR | 95% CI | P value |
| Age | 1.66 | 0.49-5.59 | 0.41 | 1.47 | 0.36-5.98 | 0.58 |
| Biochemical recurrence | 5.36 | 1.63-17.58 | 0.005 | 1.34 | 0.22-7.90 | 0.74 |
| Clinical T | 2.41 | 1.14-5.10 | 0.02 | 2.25 | 0.91-5.53 | 0.07 |
| Gleason score | 2.15 | 1.16-3.99 | 0.01 | 1.99 | 0.79-5.02 | 0.14 |
| Laterality | 1.37 | 0.62-3.03 | 0.43 | 1.71 | 0.63-4.62 | 0.28 |
| New tumor event after initial treatment | 3.47 | 1.11-10.88 | 0.03 | 1.42 | 0.30-6.60 | 0.65 |
| Pathologic T | 1.38 | 0.47-4.08 | 0.54 | 0.30 | 0.05-1.58 | 0.15 |
| Radiation therapy | 3.20 | 0.82-12.44 | 0.09 | 2.21 | 0.51-9.59 | 0.28 |
| Race | 0.54 | 0.11-2.56 | 0.44 | 0.53 | 0.10-2.65 | 0.44 |
| PSA | 13.52 | 3.38-54.06 | 0.0002 | 8.87 | 1.51-51.8 | 0.01 |

HR: Hazard Ratio; PSA: Prostate Specific Antigen
