# Supplementary material for: EpCAM as a Novel Biomarker for Survivals in Prostate Cancer Patients
Source: Front Cell Dev Biol. 2022 Apr 20;10:843604. doi: 10.3389/fcell.2022.843604 (PMC9065552; doi:10.3389/fcell.2022.843604)
Supplement: Supplementary file 3 [file Table1.DOC]

**Table S1. List of survival differential expression genes (TCGA database)**

| Gene | KM P-value | Hazard ratio | Cox P-value |
| --- | --- | --- | --- |
| ARHGEF38 | 0.007 | 2.651(1.034-6.790) | 0.0422 |
| SLPI | 0.010 | 0.675(0.470-0.968) | 0.0325 |
| EpCAM | 0.027 | 2.986(1.156-7.718) | 0.0239 |
| C1QTNF1 | 0.030 | 0.426(0.187-0.967) | 0.0412 |
| HBB | 0.033 | 0.463(0.231-0.928) | 0.0300 |

KM: Kaplan-Meier
