# Supplementary material for: EpCAM as a Novel Biomarker for Survivals in Prostate Cancer Patients
Source: Front Cell Dev Biol. 2022 Apr 20;10:843604. doi: 10.3389/fcell.2022.843604 (PMC9065552; doi:10.3389/fcell.2022.843604)
Supplement: Supplementary file 4 [file Table5.DOC]

**Table S5. Correlation between EpCAM and related gene markers of immune cells in PCa.**

| Immune cell | Gene markers | Correlation score | P-value |
| --- | --- | --- | --- |
| B cell | CD19 | -0.170 | *** |
|  | CD79A | -0.132 | ** |
| CD8+ T cell | CD8A | -0.256 | *** |
|  | CD8B | 0.006 | 0.891 |
| CD4+ T cell | CD4 | -0.222 | *** |
| M1 macrophage | NOS2 | 0.084 | 0.061 |
|  | IRF5 | -0.122 | ** |
|  | PTGS2 | -0.088 | * |
| M2 macrophage | CD163 | -0.151 | *** |
|  | VSIG4 | -0.188 | *** |
|  | MS4A4A | -0.230 | *** |
| Neutrophil | CEACAM8 | 0.023 | 0.604 |
|  | ITGAM | -0.256 | *** |
|  | CCR7 | -0.169 | *** |
| Dendritic cell | HLA-DPB1 | -0.281 | *** |
|  | HLA-DQB1 | -0.193 | *** |
|  | HLA-DRA | -0.207 | *** |
|  | HLA-DPA1 | -0.224 | *** |
|  | CD1C | -0.225 | *** |
|  | NRP1 | 0.217 | *** |
|  | ITGAX | -0.267 | *** |

*P < 0.05, **P < 0.01, ***P <0 .001
