# Supplementary material for: EpCAM as a Novel Biomarker for Survivals in Prostate Cancer Patients
Source: Front Cell Dev Biol. 2022 Apr 20;10:843604. doi: 10.3389/fcell.2022.843604 (PMC9065552; doi:10.3389/fcell.2022.843604)
Supplement: Supplementary file 5 [file Table2.DOC]

Table S2-1

Correlation between ARHGEF38 mRNA expression and clinicopathologic features in TCGA database.

| Clinical features | | ARHGEF38 expression | | P value |
| --- | --- | --- | --- | --- |
|  |  | Low (%) | High (%) |  |
| Age | ≤65 | 191(69.5) | 201(72.8) | 0.382 |
|  | >65 | 84(30.5) | 75(27.2) |  |
| Race | White | 234(85.1) | 237(85.9) | 0.263 |
|  | Black/African american | 37(13.5) | 30(10.9) |  |
|  | Asian | 4(1.5) | 9(3.3) |  |
| Laterality | Bilateral | 249(90.5) | 239(86.6) | 0.335 |
|  | Left | 9(3.3) | 14(5.1) |  |
|  | Right | 17(6.2) | 23(8.3) |  |
| Biochemical recurrence | Yes | 38(13.8) | 33(12.0) | 0.514 |
|  | No | 237(86.2) | 243(88.0) |  |
| Clinical M stage | M0 | 272(98.9) | 275(99.6) | 0.314 |
|  | M1 | 3(1.1) | 1(0.4) |  |
| Clinical T stage | T1 | 121(44.0) | 123(44.6) | 0.923 |
|  | T2 | 119(43.3) | 113(40.9) |  |
|  | T3 | 33(12.0) | 39(14.1) |  |
|  | T4 | 2(0.7) | 1(0.4) |  |
| Gleason score | 6 | 26(9.5) | 25(9.1) | 0.064 |
|  | 7 | 156(56.7) | 131(47.5) |  |
|  | 8 | 27(9.8) | 40(14.5) |  |
|  | 9 | 64(23.3) | 78(28.3) |  |
|  | 10 | 2(0.7) | 2(0.7) |  |
| New tumor event after initial treatment | YES | 38(13.8) | 61(22.1) | **0.011** |
|  | NO | 237(86.2) | 215(77.9) |  |
| Pathologic N stage | N0 | 230(83.6) | 221(80.1) | 0.278 |
|  | N1 | 45(16.4) | 55(19.9) |  |
| Pathologic T stage | T2 | 125(45.5) | 97(35.1) | **0.024** |
|  | T3 | 142(51.6) | 173(62.7) |  |
|  | T4 | 8(2.9) | 6(2.2) |  |
| Radiation therapy | YES | 29(10.5) | 48(17.4) | **0.020** |
|  | NO | 246(89.5) | 228(82.6) |  |
| Prior malignancy diagnosis | YES | 13(4.7) | 16(5.8) | 0.574 |
|  | NO | 262(95.3) | 260(94.2) |  |
| PSA | ≤10 | 265(96.4) | 265(96.0) | 0.831 |
|  | >10 | 10(3.6) | 11(4.0) |  |

Table S2-2

Correlation between C1QTNF1 mRNA expression and clinicopathologic features in TCGA database.

| Clinical features | | C1QTNF1 expression | | P value |
| --- | --- | --- | --- | --- |
|  |  | Low (%) | High (%) |  |
| Age | ≤65 | 190(69.1) | 202(73.2) | 0.289 |
|  | >65 | 85(30.9) | 74(26.8) |  |
| Race | White | 234(85.1) | 237(85.9) | 0.354 |
|  | Black/African american | 32(11.6) | 35(12.7) |  |
|  | Asian | 9(3.3) | 4(1.4) |  |
| Laterality | Bilateral | 239(86.9) | 249(90.2) | 0.474 |
|  | Left | 13(4.7) | 10(3.6) |  |
|  | Right | 23(8.4) | 17(6.2) |  |
| Biochemical recurrence | Yes | 37(13.5) | 34(12.3) | 0.691 |
|  | No | 238(86.5) | 242(87.7) |  |
| Clinical M stage | M0 | 273(99.3) | 274(99.3) | 0.997 |
|  | M1 | 2(0.7) | 2(0.7) |  |
| Clinical T stage | T1 | 112(40.7) | 132(47.8) | **0.033** |
|  | T2 | 117(42.5) | 115(41.7) |  |
|  | T3 | 44(16.0) | 28(10.1) |  |
|  | T4 | 2(0.7) | 1(0.4) |  |
| Gleason score | 6 | 24(8.7) | 27(9.8) | 0.061 |
|  | 7 | 131(47.6) | 156(56.5) |  |
|  | 8 | 42(15.3) | 25(9.1) |  |
|  | 9 | 76(27.6) | 66(23.9) |  |
|  | 10 | 2(0.7) | 2(0.7) |  |
| New tumor event after initial treatment | YES | 58(21.1) | 41(14.9) | 0.057 |
|  | NO | 217(78.9) | 235(85.1) |  |
| Pathologic N stage | N0 | 212(77.1) | 239(86.6) | **0.004** |
|  | N1 | 63(22.9) | 37(13.4) |  |
| Pathologic T stage | T2 | 100(36.4) | 122(44.2) | 0.073 |
|  | T3 | 168(61.1) | 147(53.3) |  |
|  | T4 | 7(2.5) | 7(2.5) |  |
| Radiation therapy | YES | 50(18.2) | 27(9.8) | **0.004** |
|  | NO | 225(81.8) | 249(90.2) |  |
| Prior malignancy diagnosis | YES | 20(7.3) | 9(3.3) | **0.035** |
|  | NO | 255(92.7) | 267(96.7) |  |
| PSA | ≤10 | 261(94.9) | 269(97.5) | 0.117 |
|  | >10 | 14(5.1) | 7(2.5) |  |

Table S2-3

Correlation between HBB mRNA expression and clinicopathologic features in TCGA database.

| Clinical features | | HBB expression | | P value |
| --- | --- | --- | --- | --- |
|  |  | Low (%) | High (%) |  |
| Age | ≤65 | 194(70.5) | 198(71.7) | 0.757 |
|  | >65 | 81(29.5) | 78(28.3) |  |
| Race | white | 237(86.2) | 234(84.8) | 0.207 |
|  | Black/African american | 29(10.5) | 38(13.8) |  |
|  | Asian | 9(3.3) | 4(1.5) |  |
| Laterality | Bilateral | 242(88.0) | 246(89.1) | 0.810 |
|  | Left | 13(4.7) | 10(3.6) |  |
|  | Right | 20(7.3) | 20(7.2) |  |
| Biochemical recurrence | Yes | 36(13.1) | 35(12.7) | 0.886 |
|  | No | 239(86.9) | 241(87.3) |  |
| Clinical M stage | M0 | 274(99.6) | 273(98.9) | 0.314 |
|  | M1 | 1(0.4) | 3(1.1) |  |
| Clinical T stage | T1 | 111(40.4) | 133(48.2) | **0.019** |
|  | T2 | 117(42.5) | 115(41.7) |  |
|  | T3 | 46(16.7) | 26(9.4) |  |
|  | T4 | 1(0.4) | 2(0.7) |  |
| Gleason score | 6 | 22(8.0) | 29(10.5) | **0.019** |
|  | 7 | 135(49.1) | 152(55.1) |  |
|  | 8 | 32(11.6) | 35(12.7) |  |
|  | 9 | 84(30.5) | 58(21.0) |  |
|  | 10 | 2(0.7) | 2(0.7) |  |
| New tumor event after initial treatment | YES | 57(20.7) | 42(15.2) | 0.092 |
|  | NO | 218(79.3) | 234(84.8) |  |
| Pathologic N stage | N0 | 218(79.3) | 233(84.4) | 0.117 |
|  | N1 | 57(20.7) | 43(15.6) |  |
| Pathologic T stage | T2 | 95(34.5) | 127(46.0) | **0.002** |
|  | T3 | 169(61.5) | 146(52.9) |  |
|  | T4 | 11(4.0) | 3(1.1) |  |
| Radiation therapy | YES | 48(17.4) | 29(10.5) | **0.019** |
|  | NO | 227(82.5) | 247(89.5) |  |
| Prior malignancy diagnosis | YES | 14(5.1) | 15(5.4) | 0.857 |
|  | NO | 261(94.9) | 261(94.6) |  |
| PSA | ≤10 | 268(97.5) | 262(94.9) | 0.121 |
|  | >10 | 7(2.5) | 14(5.1) |  |

Table S2-4

Correlation between SLPI mRNA expression and clinicopathologic features in TCGA database.

| Clinical features | | SLPI expression | | P value |
| --- | --- | --- | --- | --- |
|  |  | Low (%) | High (%) |  |
| Age | ≤65 | 194(70.5) | 198(71.7) | 0.757 |
|  | >65 | 81(29.5) | 78(28.3) |  |
| Race | White | 225(81.8) | 246(89.1) | **0.041** |
|  | Black/African american | 43(15.6) | 24(8.7) |  |
|  | Asian | 7(2.5) | 6(2.2) |  |
| Laterality | Bilateral | 242(88.0) | 246(89.1) | 0.916 |
|  | Left | 12(4.4) | 11(4.0) |  |
|  | Right | 21(7.6) | 19(6.9) |  |
| Biochemical recurrence | Yes | 39(14.2) | 32(11.6) | 0.365 |
|  | No | 236(85.8) | 244(88.4) |  |
| Clinical M stage | M0 | 274(99.6) | 273(98.9) | 0.317 |
|  | M1 | 1(0.4) | 3(1.1) |  |
| Clinical T stage | T1 | 128(46.5) | 116(42.0) | 0.923 |
|  | T2 | 103(37.5) | 129(46.7) |  |
|  | T3 | 43(15.6) | 29(10.5) |  |
|  | T4 | 1(0.4) | 2(0.7) |  |
| Gleason score | 6 | 33(12.0) | 18(6.5) | 0.064 |
|  | 7 | 138(50.2) | 149(54.0) |  |
|  | 8 | 32(11.6) | 35(12.7) |  |
|  | 9 | 69(25.1) | 73(26.4) |  |
|  | 10 | 3(1.1) | 1(0.4) |  |
| New tumor event after initial treatment | YES | 46(16.7) | 53(19.2) | 0.449 |
|  | NO | 229(83.3) | 223(80.8) |  |
| Pathologic N stage | N0 | 231(84.0) | 220(79.7) | 0.191 |
|  | N1 | 44(16.0) | 56(20.3) |  |
| Pathologic T stage | T2 | 117(42.5) | 105(38.0) | **0.024** |
|  | T3 | 152(55.3) | 163(59.1) |  |
|  | T4 | 6(2.2) | 8(2.9) |  |
| Radiation therapy | YES | 34(12.4) | 43(15.6) | 0.276 |
|  | NO | 241(87.6) | 233(84.4) |  |
| Prior malignancy diagnosis | YES | 18(6.5) | 11(4.0) | 0.178 |
|  | NO | 257(93.5) | 265(96.0) |  |
| PSA | ≤10 | 265(96.4) | 265(96.0) | 0.831 |
|  | >10 | 10(3.6) | 11(4.0) |  |
